# Tandem combination of ASCT and CAR T‐cell therapy in highly refractory CNS lymphomas

**DOI:** 10.1111/bjh.70132

**Published:** 2025-08-31

**Authors:** Lydia Montes, Sylvain Choquet, Madalina Uzunov, Véronique Morel, Marine Baron, Damien Roos‐Weil, Magalie Joris, Caroline Delette, Delphine Lebon, Nabih Azar, Carole Metz, Lucia Nichelli, Magali Le Garff‐Tavernier, Clémentine Boccon‐Gibod, Claire Lacan, Laetitia Souchet, Khê Hoang‐Xuan, Carole Soussain, Inès Boussen, Caroline Houillier

**Affiliations:** ^1^ Hematology Department CHU Amiens Amiens France; ^2^ Hematology Department APHP Pitié Salpétrière Paris France; ^3^ Internal Medicine APHP Pitié Salpétrière Paris France; ^4^ Pharmacy Department APHP Pitié Salpétrière Paris France; ^5^ Neuro‐Radiology Department APHP Pitié Salpétrière Paris France; ^6^ Hematology Biology Department APHP Pitié Salpétrière Paris France; ^7^ Onco‐Neurology Department APHP Pitié Salpétrière Paris France; ^8^ Hematology Department Institut Curie Paris France

**Keywords:** autologous stem cell transplantation, CART cells, chemotherapy, lymphoma

To the Editor,

In patients eligible with central nervous system (CNS) lymphoma, the standard treatment consists of induction with high‐dose methotrexate (HD‐MTX) and then, if the response is good, consolidation with thiotepa‐based high‐dose chemotherapy and autologous stem cell transplantation (ASCT).^1^, ^2^ The prognosis after this treatment is rather good, with 5‐year progression‐free survival (PFS) and overall survival (OS) rates of 74% and 80% respectively.^3^ However, 25%–30% of the patients are refractory to HD‐MTX^4^ and need salvage treatment followed by ASCT. Schenone et al. have shown that the patients' prognosis after ASCT was closely associated with the number of lines of treatment prior to ASCT.^3^ The 1‐year PFS rate was around 80% for first‐line treatment, around 70% for second‐line treatment and around 50% for subsequent lines of treatment; this latter value is rather disappointing.

Anti‐CD19 chimeric antigenic receptor (CAR) T cells represent one of the last decade's major improvements in the treatment of large B‐cell lymphoma (LBCL).^5^, ^6^ Whereas CNS lymphomas were initially excluded from the trials on CAR T cells because of the high expected risk of neurotoxicity, several recent studies have highlighted an acceptable safety profile and promising efficacy results in highly pretreated patients.^7^, ^8^ However, the relapse rate following CAR T‐cell therapy was still high, and the 1‐year PFS rate did not exceed 40%–45%.^7^, ^8^

Furthermore, disease progression prior to ASCT or CAR T‐cell treatment is known to be a major factor in the failure of these approaches.^1^, ^7^, ^9^

The treatment of young patients with highly refractory CNS lymphomas must therefore be improved. Since 2023, the French expert network on CNS lymphomas (Lymphomes Oculo‐Cérébraux (LOC) network) has considered in this setting a regimen combining ASCT directly followed a several weeks later by CAR T‐cell therapy. The aim of this study was to evaluate the efficacy and tolerance of this strategy.

We retrospectively retrieved from the LOC network database the adult patients for whom the intent‐to‐treat project of combination of ASCT and CAR T cells was considered in the context of a refractory CNS LBCL, either primary or secondary, without any systemic lesions at the time of the decision. The database was approved by the Institutional Ethical Committee of the coordinating centre and the French National Data Protection Commission. The medical charts of the patients were also reviewed for detailed information. All patients gave their written informed consent to the collection and use of their personal data.

The treatment response was assessed by reference to the International Primary CNS Lymphoma Collaborative Group criteria.^10^ Cytokine release syndrome (CRS) and immune effector cell‐associated neurotoxicity syndrome (ICANS) were graded according to the autologous stem cell transplantation (ASTCT) 2019 guidelines.^11^ Other adverse events were documented with regard to the Common Terminology Criteria for Adverse Events (version 5.0). The PFS and OS were calculated from the date of ASCT. Survival rates were calculated using the Kaplan–Meier method. All statistical analyses were carried out with R software.

Five patients met the study's inclusion criteria (Table 1 and Table S1). The median number of previous lines of treatment was 3 (range: 3–5); these included HD‐MTX, high‐dose cytarabine, ifosfamide–carboplatin–etoposide and rituximab in all patients. All patients were refractory to first‐line treatment and had undergone lymphapheresis prior to ASCT. At the time of ASCT, the median age was 39 (range: 30–53), and the median Karnofsky performance status (KPS) was 70 (range: 60–90); three patients had progressive disease, one had a partial response (PR) and one had a complete response (CR). Four of the five patients received a thiotepa–busulfan–cyclophosphamide regimen, and the fifth received a thiotepa–carmustine regimen. Although the level of post‐ASCT toxicity was acceptable in four patients, one patient presented several severe infectious complications and severe bladder bleeding, and we therefore decided not to administer the CAR T cells (Table 2). This patient relapsed (with both systemic and CNS lesions) 8 months after ASCT and received several courses of salvage treatment, including CAR T‐cell therapy 12 months after ASCT. Nineteen months after ASCT, he is alive with a CR, but he developed pancytopenia secondary to a ring sideroblastic myelodysplasia with a monosomy 7. In the four remaining patients, the median time interval between ASCT and CAR T‐cell infusion was 75 days (range: 67–86). None of the patients received any other cancer treatments in the time interval between ASCT and CAR T‐cell therapy. At the time of CAR T‐cell infusion, the median KPS was 70 (range: 60–80), three patients showed a CR and one patient showed a PR. All the patients received axicabtagene ciloleucel. One patient experienced a neurological adverse event (grade 3 ICANS), and four experienced grade 1 CRS. Three patients presented with thrombocytopenia and neutropenia grade ≥3 lasting for more than 28 days after CAR T‐cell therapy. Three months after CAR T‐cell infusion, two patients still presented grade 3 thrombocytopenia, and one presented grade 3 neutropenia. Two patients were readmitted to hospital within 6 months of the CAR T‐cell therapy due to sepsis (Table 2).

**TABLE 1:** Patients' characteristics.

*N* at ASCT/N at CAR T cells	5/4
Sex ratio (M/W)	5/0
Immunocompetent status at initial diagnosis	5
Initial diagnosis	
PCSNL	3
Systemic + CNS DLBCL	1
Systemic DLBCL	1
Refractory to first‐line treatment	5
Duration of disease before ASCT: median (range) (months)	13 (8–139)
Number of lines of treatment before ASCT: median (range)	3 (3–5)
Previous treatments	
HD‐MTX	5
HD‐AraC	5
ICE	5
Lenalidomide	3
Ibrutinib	4
Rituximab	5
WBRT	2
Leukapheresis before ASCT	5
Disease status at ASCT	
PD	3
PR	1
CR	1
Age at ASCT: median (range)	39 (30–53)
KPS at ASCT: median (range)	70 (60–90)
HCT regimen	
TBC	4
Thiotepa‐carmustine	1
Other therapies between ASCT and CAR T cells	0
Delay between leukapheresis and CAR T cells: median (range) (days)	82 (73–97)
Delay between ASCT and CAR T cells: median (range) (days)	75 (67–86)
Disease status at CAR T‐cell infusion	
PR	1
CR	3
KPS at CAR T‐cell infusion: median (range)	70 (60–80)
Type of myeloablative CT before CAR T cells	
Fludarabine–cyclophosphamide	4
Type of CAR T cells	
Axi‐cel	4
Best response after CAR T cells	
CR	4
Relapse after CAR T cells	0
Other therapy after CAR T cells	0
Follow‐up from ASCT: median (min–max) (months)	18 (14–22)
1‐year PFS: % (95% CI)	80 (52–100)
1‐year OS: %	100

Abbreviations: ASCT, high‐dose chemotherapy with autologous stem cell transplantation; axi‐cel, axicabtagene ciloleucel; CAR T‐cells, chimeric antigen receptor T cells; CNS, central nervous system; CR, complete response; CT, chemotherapy; DLBCL, diffuse large B‐cell lymphoma; HCT, high‐dose chemotherapy; HD‐AraC, high‐dose cytarabine; HD‐MTX, high‐dose methotrexate; ICE, ifosfamide–carboplatin–etoposide; KPS, Karnofsky performance status; M, men; *N*, number; OS, overall survival; PCNSL, primary central nervous system lymphoma; PD, progressive disease; PFS, progression‐free survival; PR, partial response; TBC, thiotepa–busulfan–cyclophosphamide; W, women; WBRT, whole‐brain radiotherapy.

**TABLE 2:** Profile of tolerance and main toxicities after ASCT and CAR T cells.

ASCT (*N* = 5)	
Febrile neutropenia following ASCT	5
Duration of grade 4 neutropenia after ASCT (days): median (min–max)	12 (8–20)
Duration of grade 4 thrombopenia after ASCT (days): median (min–max)	9 (5–15)
Grade 3–4 hepatotoxicity after ASCT	1
Grade 3–4 nephrotoxicity after ASCT	1
Transfer in ICU after ASCT	2
Duration of hospitalization for ASCT (days): median (min‐max)	32 (19–136)
Toxic death after ASCT	0
CAR T cells (*N* = 4)	
CRS	4
Including grade 3–4 CRS	0
ICANS	1
Including grade 3–4 ICANS	1
Grade 3–4 hepatotoxicity after CAR T cells	0
Grade 3–4 nephrotoxicity after CAR T cells	0
Transfer in ICU after CAR T cells	1
Duration of hospitalization for CAR T cells (days): median (min–max)	26 (22–29)
Cytopenia grade ≥3 lasting more than 28 days/3 months/6 months after CAR T cells	
Thrombopenia	3/2/0
Neutropenia	3/1/0
Anaemia	0/0/0
Hypogammaglobulinaemia <4 g/L lasting more than 6 months/12 months	2/1
Number of subsequent rehospitalizations during the 6 months following CAR T cells and causes	4 SARS‐COV2 with surinfection (ICU) Colitis and pancreatitis with hypovolemic shock (ICU) Nausea/vomiting Femoral neck fracture
Toxic death after CAR T cells	0

Abbreviations: ASCT, high‐dose chemotherapy with autologous stem cell transplantation; CRS, cytokine release syndrome; ICANS, immune effector‐cell associated neurotoxicity syndrome; ICU, intensive care unit; *N*, number.

All four patients achieved a CR after CAR T‐cell therapy. None relapsed or received any subsequent cancer treatments. All four were alive after a median follow‐up period of 18 months (range: 14–22 months) after ASCT (median follow‐up period of 15 months; range: 12–19 months) from CAR T cells. At last follow‐up, the median (range) KPS was 80 (range: 70–90), and all four patients were living at home. Three of the four had returned to work, whereas a return was prevented by the fourth patient's neurological sequelae due to lymphoma.

A combination of ASCT and CAR T‐cell therapy has already been reported in patients with poor‐risk diffuse LBCL^12^, ^13^ or multiple myeloma.^14^ In these studies, the CAR T cells were infused a few days after ASCT. The rationale was that myeloablative ASCT might increase the effectiveness of adoptive T‐cell immunotherapy, notably by acting on the immune microenvironment. Thus, Wei et al. reported better outcomes in patients with relapsing/refractory LBCL with TP53 alteration who underwent ASCT and treatment with a cocktail of anti‐CD19 and anti CD22 CAR T cells, relative to patients who were treated with CAR‐T cells only.

In the field of CNS lymphoma, Yu et al. reported on a series of 22 patients: Nine had undergone ASCT and then, a few days later, CAR T‐cell therapy.^9^ However, it is difficult to draw clear conclusions from these results because Yu et al. did not explain why some patients received the combination and others did not. Moreover, most patients were also treated with various drugs (notably anti‐PD‐1 agents and Bruton tyrosine kinase inhibitors), and some of the patients were conditioned with a carmustine, etoposide, cytarabine, melphalan (BEAM) regimen, which is not optimal in the context of CNS lymphoma. Data on toxicity following the combination were limited.

Our rationale was to try to reduce the tumour burden with ASCT as much as possible, in order to improve the response to CAR T‐cell therapy and limit the neurotoxicity induced by the CAR T‐cell procedure. In our network, we chose to administer CAR T cells several days after ASCT (median of 75 days). This time interval (i) reduced the likelihood of severe adverse events because thiotepa‐based conditioning regimens are known to be more toxic than BEAM regimens, and (ii) allowed us to control the tumour reduction burden before CAR T‐cell therapy. Moreover, most patients required emergency ASCT, and the CAR T‐cells were not ready at the time of transplantation. Similar strategies have been reported in acute leukaemia, with a combination of chemotherapies and allogeneic transplantation.^15^

All the patients in our series had several risk factors for a poor prognosis. They had all been treated extensively prior to ASCT and had highly refractory CNS lymphomas. All were refractory to first‐line treatment, and three displayed progressive disease at the time of ASCT. Despite the presence of these factors, the four patients who received the combination of ASCT and CAR T‐cell therapy achieved a CR that has lasted for at least 12 months after CAR T cells, in the absence of other cancer treatment—a very good result in this setting. The level of toxicity observed after the combined treatment appeared to be acceptable in this population of young adult patients in good general condition. Notably, there was only one case of grade 3 neurotoxicity. The level of haematological toxicity after CAR T‐cell therapy appeared to be higher than that typically reported in the literature (two patients were readmitted to hospital for infections), but the latter had resolved in all patients within 6 months of CAR T‐cell therapy. However, to determine the risk of myelodysplasia associated with this combination treatment, studies with the prolonged follow‐up of a larger number of patients will be needed. At last follow‐up, all the patients had a reasonable quality of life.

The present results are preliminary and must be confirmed in larger series with a longer follow‐up period. Nevertheless, the combination of ASCT with CAR T‐cell therapy already appears to be a valuable option in young adult patients suffering from highly refractory CNS lymphomas.

## AUTHOR CONTRIBUTIONS

LM and CH: Acquisition and analysis of data; writing of the manuscript. SC, MU, VM, MB, DRW, MJ, CD, DL, NA, CM, LN, MLGT, CBG, CL, LS, KHX, CS and IB: Acquisition of data; review of the manuscript and approval of the final version.

## CONFLICT OF INTEREST STATEMENT

Sylvain Choquet: Novartis, Kite/gilead. Other authors: None.

## Supporting information


Table S1.

## Data Availability

The data that support the findings of this study are available from the corresponding author upon reasonable request.
