# Supplementary material for: Tandem combination of ASCT and CAR T‐cell therapy in highly refractory CNS lymphomas
Source: Br J Haematol. 2025 Aug 31;207(5):2178–82. doi: 10.1111/bjh.70132 (PMC12624162; doi:10.1111/bjh.70132)
Supplement: Supplementary file 1 — Table S1. [file BJH-207-2178-s001.docx]

Supplementary Table 1 : characteristics of each patient

| Patient | Initial diagnosis | Previous treatments and response | Duration of disease before ASCT (months) | Age at ASCT | KPS at ASCT | Tumoral status at ASCT | HCT regimen | Duration of grade 4 neutropenia/ thrombopenia after ASCT | Febrile aplasia after ASCT | Transfer in ICU after ASCT | Duration of hospitalization for ASCT (days) | Delay between ASCT and CAR T-cells (days) |
| --- | --- | --- | --- | --- | --- | --- | --- | --- | --- | --- | --- | --- |
| 1 | PCNSL | L1 : R-MPV-lenalidomide + R-cytarabine -> PD  L2 : R-ICE -> PD  L3 : IT MTX + lenalidomide + ibrutinib -> CR | 8 | 31 | 60 | CR | TBC | 14/6 | Yes | No | 32 | 74 |
| 2 | PCNSL | L1 : R-MPV-ibrutinib + R-cytarabine -> PD  L2 : R-ICE -> PD  L3 : lenalidomide + ibrutinib -> PD | 12 | 39 | 70 | PD | TBC | 12/5 | Yes | Yes | 45 | 77 |
| 3* | Systemic + CNS DLBCL | L1 : R-CHOP-MTX alternating with R-MTX-cytarabine -> PD  L2 : R-ICE -> PD  L3 : ibrutinib -> PD | 13 | 47 | 90 | PD | TBC | 20/15 | Yes | Yes | 136 | NA |
| 4 | PCNSL | L1 : R-MBVP + R-AraC -> PD  L2 : WBRT -> CR  L3 : R-ICE -> PD  L4 : lenalidomide+ ibrutinib -> PD  L5 : pomalidomide + ibrutinib -> PD | 139 | 53 | 70 | PD | TBC | 9/9 | Yes | No | 31 | 86 |
| 5 | Systemic DLBCL | L1 : R-ACBVP + MTX IT, R-ifosfamide-VP16 -> PD  L2 : Matrix -> PD  L3 : R-ICE -> PD  L4 : WBRT -> PR | 14 | 30 | 90 | PR | TTP-BCNU | 8/12 | Yes | No | 19 | 67 |

PCNSL : primitive central nervous system lymphoma, DLBCL : diffuse large B cell lymphoma, RMPV : Rituximab Methotrexate Procarbazine Vincristine, ICE : Ifosfamide Carboplatine Etoposide, CHOP : cyclophosphamide Hydroxyadriamycine Onconvin prednison, PD : progressive disease, PR : partial response, CR : complete response, ASCT : Autologous stem cell transplantation, HCT : High condition therapy, TBC : Thiotepa Busulfan cyclophosphamide

*Patient 3 didn’t receive CAR T-cells following ASCT because of an important ASCT-related toxicity

| Patient | Tumoral status at CAR T-cell infusion | KPS at CAR T-cell infusion | Type of CAR T-cells | CRS grade | ICANS grade | Transfer in ICU after ASCT | Grade ≥3 cytopenia lasting >28 days after CAR T-cells | Best response after CAR T-cells | Subsequent relapse(s) | Antitumoral treatment after ASCT/CAR T-cells | KPS at latest news | PFS after ASCT | OS after ASCT |
| --- | --- | --- | --- | --- | --- | --- | --- | --- | --- | --- | --- | --- | --- |
| 1 | CR | 70 | Axi-cel | 1 | 0 | No | No | CR | No | No | 80 | 18+ | 18+ |
| 2 | PR | 70 | Axi-cel | 1 | 0 | No | Neutropenia, thrombopenia | CR | No | No | 80 | 22+ | 22+ |
| 3* | NA | NA | NA | NA | NA | NA | NA | NA | Yes | IT R-MTX + epco-lena +  axi-cel | 90 | 8 | 19+ |
| 4 | CR | 60 | Axi-cel | 1 | 3 | Yes | Neutropenia, thrombopenia | CR | No | No | 70 | 16+ | 16+ |
| 5 | CR | 80 | Axi-cel | 1 | 1 | No | Neutropenia, thrombopenia | CR | No | No | 90 | 14+ | 14+ |
